# Severe sepsis caused by serious gastrointestinal infection in sJIA patients treated with IL-6 receptor antagonist: a case report

**DOI:** 10.1186/s12887-020-02032-w

**Published:** 2020-03-18

**Authors:** Meng Xu, Congcong Liu, Lishuang Guo, Sirui Yang

**Affiliations:** Department of Pediatric Rheumatology, Immunology and Allergy, The First Hospital of Jilin University, Changchun, 130021 China

**Keywords:** Systemic juvenile idiopathic arthritis, Tocilizumab, Gastrointestinal infection, Sepsis, Case report

## Abstract

**Background:**

Interleukin (IL)-6 plays an essential role in the pathogenesis of systemic juvenile idiopathic arthritis (sJIA). Tocilizumab (TCZ), a kind of biological agent against both membrane and soluble IL-6 receptor, is the only biological agent approved for the treatment of sJIA in China. Infections are the most common adverse events during TCZ therapy, and most of infections are mild or moderate. Severe sepsis originated from gastrointestinal infection is rarely reported.

**Case presentation:**

In this article, we reported two 13-year-old sJIA patients who suffered from life-threatening infections after TCZ administration. Within one day, both of them presented rapidly progressive conditions that included fever, abdominal pain, dizziness, diarrhea and vomiting, and laboratory tests showed multi-organ dysfunctions. They were diagnosed with severe sepsis and septic shock that were supposed to be caused by the pathogens from the gastrointestinal tract, and they were eventually rescued by timely treatment. In addition, we also reviewed the literature about serious gastrointestinal infections and sepsis in sJIA patients receiving TCZ therapy.

**Conclusions:**

In summary, for sJIA patients with TCZ therapy, invading pathogens from the gastrointestinal tract can cause an intensely systemic infection that may even be fatal. Therefore, it is essential to pay attention to the gastrointestinal management of sJIA patients as well as remind them of their intestinal hygiene.

## Background

Systemic juvenile idiopathic arthritis (sJIA) is an auto-inflammatory disease characterized by high spiking fever, evanescent skin rash, arthritis, and remarkable overexpression of pro-inflammatory cytokines, such as interleukin (IL)-1, IL-6, and tumor growth factor-alpha (TNF-α) [1]. Biological agents that target these cytokines or their receptors can efficiently delay the joint damage. Tocilizumab (TCZ) is an IL-6 receptor antagonist binding to both membrane-bound and soluble IL-6 receptors; it is increasingly used for sJIA treatment in China. Notably, infections are the main adverse events [2], and may also be masked [3, 4]. sJIA patients treated with TCZ are susceptible to infections, especially respiratory and gastrointestinal (GI) [5]. However, severe sepsis caused by GI infections is rarely recorded. Herein, we reported two cases of sJIA patients who suffered from severe sepsis caused by GI infections and also reviewed the literature regarding sepsis and GI infections occurring in sJIA patients receiving TCZ therapy.

## Case presentation

### Case 1

A 13-year-old boy was admitted to our hospital in the fall of 2017, with the complaint of persistent fever, abdominal pain, dizziness, diarrhea, and vomiting for one day. During the past day, the temperature ranged from 38.6 to 40.2 °C. Before disease onset, he ate some underdone pork. In contrast to his severe condition, his parents, who ate the same, just exhibited mild diarrhea. At admission, the patient presented signs of shock, including unconsciousness, weak pulse, irregular heart and respiratory rhythm, undetectable blood pressure, and prolonged capillary refilling time (CRT). Therefore, he was transferred to the pediatric intensive care unit (PICU). The white blood cell count (WBC) was 20.41 × 10^9^/L, and the value of C-reactive protein (CRP) was 107.13 mg/L (Table 1). Laboratory tests also indicated the dysfunction of the heart, liver, and kidney. Bedside X-ray excluded the perforation and obstruction of the GI tract. The abdominal ultrasound found a few liquid-like intestinal contents. He was diagnosed with severe sepsis and septic shock; rescue strategies, including fluid resuscitation, vasopressor agents, and mechanical ventilation, were performed. Additionally, continuous renal replacement therapy and intravenous meropenem, methylprednisolone (2 mg/kg twice per day), and intravenous immunoglobulins (IVIG, 400 mg/kg per day for three days) were administered because of the low level of IgG. On the third day of hospitalization, blood culture detected *Salmonella enteritidis*, which was sensitive to meropenem. Fourteen days after the admission, he was transferred to our department with a stable condition. Subsequently, TCZ was removed from the therapeutic plan.

**Table 1:** Clinical and laboratory characteristics of the two patients

	Case 1	Case 2	
General characteristics			
Age, years	13	13	
Sex	Male	Male	
Weight, kg	40.7	54	
Dose of TCZ	8 mg/kg	8 mg/kg	
Potential cause	Underdone pork	Roast meat	
Initial symptoms	Fever	Fever	
	Abdominal pain	Abdominal pain	
	Dizziness	Dizziness	
	Diarrhea	Diarrhea	
	Vomiting	Vomiting	
Signs			
State	Unconsciousness	Dysphoria	
HR, /min	141	174	
RR, /min	41	56	
BP, mmHg	Undetectable	80/42	
MAP, mmHg	Undetectable^a^	56^a^	
SpO_2_, %	76	83	
CRT, second	5	> 5	
Laboratory findings			Normal value
WBC, 10^9^/L	20.41	38.76	3.5–9.5
Neutrophil, %	78	87	40–75
Hemoglobin, g/L	118	121	115–150
Platelet, 10^9^/L	458	399	125–350
CRP, mg/L	107.13	95.9	0–3
PCT, ng/ml	15.86	37.19	0–0.5
ESR, mm/h	113	128	0–20
Ferritin, μg/L	4118^b^	5560^b^	10–120
Fibrinogen, g/L	2.6^b^	3.01^b^	1.8–4.0
APTT, second	61.7	31.2	21–33
PT, second	29	17.3	0–13
AST, U/L	71.4^b^	238.1^b^	13–35
ALT, U/L	94.3	982.3	7–40
BNP, pg/ml	17,800	1230	0.1–1.0
Creatinine, μmol/L	305.4^a^	295.3^a^	41–73
Lactic acid, mmol/L	5.3	4.7	0–125
IgG, g/L	3.98	6.8	8.6–17.4
IgA, g/L	0.54	0.63	1.0–4.2
IgM, g/L	0.32	1.3	0.5–2.8
Blood culture	Salmonella enteritidis	Negative	Negative

*HR* heart rate, *RR* respiratory rate, *BP* blood pressure, *MAP* mean arterial pressure, *SpO*_*2*_ percutaneous blood oxygen saturation, *CRT* capillary refilling time, *WBC* white blood cell counts, *CRP* C-reactive protein, *PCT* procalcitonin, *ESR* erythrocyte sedimentation rate, *APTT* activated partial thromboplastin time, *PT* prothrombin time, *AST* aspartate transaminase, *ALT* alanine transaminase, *BNP* brain natriuretic peptide, *Ig* immunoglobulin

^a^ Terms included in the criteria of sepsis

^b^ Terms included in the criteria of MAS

Seventy-six months before the admission, the patient was diagnosed with sJIA in another hospital. Initially, he was treated with intravenous methylprednisolone, oral prednisone acetate, and methotrexate (MTX). However, shortly after each cessation of the oral prednisone acetate, the disease flared with the presence of fever, skin rash, and arthritis. Four months before this admission, the patient came to our department. Considering the poor treatment response of the patient to glucocorticoid, we stopped using glucocorticoid, and then we administered the first infusion of TCZ (8 mg/kg, every two weeks for the first two months and every three weeks subsequently) and oral MTX. The last infusion of TCZ was used 13 days before this admission. During the period of TCZ therapy, there were no signs of infection and neutropenia. The laboratory tests, including liver function, renal function, and levels of immunoglobulins before each administration of TCZ were normal.

### Case 2

A 13-year-old boy was admitted to our department in the summer of 2018 due to persistent fever, abdominal pain, diarrhea, vomiting, and dizziness for one day after eating roast meat. During the past day, the minimum body temperature was 38.3 °C, and the maximum was 39.8 °C. Conversely, his family, who ate the same food, did not show any discomfort. At admission, he presented a poor condition with dysphoria, muffled heart sounds, increased heart and breath rate, low blood pressure, and prolonged CRT. Due to the unstable vital signs, he was transferred to PICU. Fluid resuscitation, vasopressor agents, and mechanical ventilation were performed immediately. The WBC was 38.76 × 10^9^/L, and the value of CRP was 95.9 mg/L (Table 1). Other laboratory tests showed multi-organ dysfunction. Bedside imaging examinations detected gas in the intestine. Also, he was diagnosed with severe sepsis and septic shock, and subsequently, meropenem, methylprednisolone (2 mg/kg twice per day), and continuous renal replacement therapy were administered. Blood culture was negative. Eleven days after admission, with the improvement of the disease, he was transferred to our department. Then, TCZ was discontinued because of this severe event. He was diagnosed with sJIA 53 months before this admission, and 20 months ago, the treatment regimen was changed from oral prednisone acetate to intravenous TCZ. Eight months before this admission, he was diagnosed with influenza with the presence of neutropenia, which returned to normal after oral oseltamivir. Thirteen days before this admission, there were no signs of infection and neutropenia, and therefore, the last infusion of TCZ was given.

#### Search strategy

The literature review was conducted to identify clinical studies regarding the serious adverse events (SAEs) of TCZ treatment in sJIA patients. A search of PubMed, without limitation on dates, with a combination of “Tocilizumab” AND “systemic juvenile idiopathic arthritis,” and with the restriction on the English language, and age for the child (birth to 18 years), was performed. Of the 65 searched articles, 21 review articles were excluded. The remaining 44 articles were reviewed; of these, 9 recorded the SAEs of TCZ therapy in sJIA patients. Information related to total SAEs, severe and GI infections and sepsis was collected.

## Results

Table 1 shows the clinical and laboratory characteristics of both patients. Table 2 summarizes the SAEs recorded by previous studies after TCZ infusion in sJIA patients [2, 6–13]. 6/9 articles recorded severe infectious events and four observed severe GI infections. In addition, two articles documented four septic events in sJIA patients receiving TCZ.

**Table 2:** Serious adverse events recorded by previous studies after TCZ infusion in sJIA patients

Authors and references	Years	Study design	Duration	No. of patients	SAEs no. of events	Serious infections no. of events	Serious GI infections no. of events	sepsis no. of events
Yokota et al. [6]	2005	OL, Phase II	14 weeks	11	0	0	0	0
Woo et al. [7]	2005	OL, Phase II	4–8 weeks	15	5	2	0	0
Yokota et al. [8]	2008	R, DB, PC, Phase III	6 weeks OL lead-in phase 12 weeks DB phase 48 weeks OL- extension phase	56	15	Unknown^b^	Unknown^b^	0
De Benedetti et al. [2]	2012	R, DB, PC, Phase III	12 weeks DB phase 96 weeks OL extension phase	112	39	18	5	1
Yokota et al. [9]	2014	Long-term extension study of their 2 previous studies	168 weeks	67	78	30	13	0
Yokota et al. [10]	2016	Post marketing surveillance	52 weeks	417	222	74	9	3
Horneff et al. [11]	2017	Retrospective study	2000–2015	71^a^	14	2	0	0
Kimura et al. [12]	2017	Pilot study	9 months	10^a^	1	0	0	0
Bielak et al. [13]	2018	Retrospective study	7/2009–4/2014	46	2	0	0	0

*OL* open-label, *R* randomized, *DB* double blind, *PC* placebo-controlled, *SAEs* serious adverse events, *GI* gastrointestinal

^a^number of patients involved in the sJIA group of the study

^b^the article did not clearly list the number of serious infection or serious GI infection, but only indicated that 2 cases of serious GI infections resolved with antibiotic treatment

## Discussion and conclusion

The two patients presented extremely similar clinical and laboratory manifestations, including fever, abdominal pain, diarrhea, vomiting, dizziness, high level of inflammatory indicators, and multi-organ dysfunctions. In addition, the vital signs were unstable at the time of admission. According to the criteria of “sepsis-3,” they were diagnosed with severe sepsis and septic shock [14]. In a randomized, double-blind, placebo-controlled Phase III trial that was conducted by De Benedetti et al. [2], one patient was diagnosed with sepsis and died on the same day. Postmortem blood and stool cultures yielded Streptococcus. The other three septic events were recorded by Yokota et al. [10] in 2015, and 1/3 patients died due to Pseudomonas infection. In the current two patients, given the history of eating unclean food and their obvious GI symptoms, we speculated that the severe illnesses were caused by GI pathogens. These GI infections are a cause of sepsis and could be fatal if the patients are not treated promptly. Also, the two patients presented remarkably similar clinical features and both developed infectious symptoms on the 13th day after TCZ administration. A correlation between the onset of severe infections and the pharmacokinetics of TCZ is yet to be elucidated. Before the two serious events, GI infection was neglected; however, now, we speculated that TCZ therapy should be recommended to sJIA patients with caution before their dietary hygiene is essential.

Given the immunosuppressive action of TCZ, we speculated that such serious illnesses resulting from GI infections were related to the blockade of IL-6 signal. The exact mechanism of TCZ related to GI infection is yet unknown. A recent study found that sJIA patients presented intestinal microbiota dysbiosis, which was partially restored in inactive patients [15]. The changes in the composition of intestinal microbiota might increase intestinal permeability [16]. Hence, the aberrant intestinal microbiota may be a cause of GI infections in the patients. Supposedly, IL-6 regulates the barrier function of GI mucous. A previous mouse-model study found that the administration of IL-6 increases intestinal hyperplasia and improves the barrier function of the small bowel. Conversely, IL-6-null mice show increased injury of the intestinal cell [17]. Thus, we speculated that inhibition of the IL-6 signal by TCZ facilitates the invasion of infectious agents through the GI tract.

Macrophage activation syndrome (MAS) is another life-threatening complication during sJIA and can be characterized by sustained fever, hyperferritinemia, cytopenia, coagulopathy, and multi-organ dysfunctions [18, 19], which are part of the features of sepsis [20]. Interestingly, clinical and laboratory features of our patients suggested MAS, including persistent fever, elevated levels of ferritin and AST, and decreased fibrinogen [21]. Since the main principle of therapy is different [22, 23], the differential diagnosis between MAS and sepsis is required, but may be difficult. However, the decrease in the number of white blood cells and platelets is the early event of MAS and often occurs before the deterioration of the patients’ conditions. Although some of the clinical features of MAS may be masked by the application of TCZ [24], leukocytopenia and thrombocytopenia are commonly observed in MAS rather than sepsis [25, 26]. In addition, MAS commonly results in the dysfunction of the central nervous system, heart, lung, and kidney, while rarely causing abdominal symptoms [27]. Based on the above considerations, the patients were diagnosed with sepsis. However, the way of differential diagnosis between sepsis and MAS could not be addressed in this case study, and more rigorous studies are needed.

In summary, invading pathogens from GI tract can lead to severe sepsis and may even be fatal if the patient is not timely treated. Therefore, clinicians should pay enough attention to gastrointestinal infections of sJIA patients who are receiving TCZ therapy. It is necessary to remind the patients to keep a clean and healthy diet.

## Data Availability

Not applicable.

## Abbreviations

CRT: Capillary refilling time
GI: Gastrointestinal
IVIG: Intravenous immunoglobulin
MAS: Macrophage activation syndrome
PICU: Pediatric intensive care unit
SAEs: Serious adverse events
sJIA: systemic-onset juvenile idiopathic arthritis
TCZ: Tocilizumab
